# Encéphalopathie de Wernicke: complication rare de l’hyperemesis gravidarum

**DOI:** 10.11604/pamj.2020.36.267.18468

**Published:** 2020-08-11

**Authors:** Soumaya Nasri, Narjisse Aichouni, Mounia Ettayeb, Yassine Mebrouk, Imane Kamaoui

**Affiliations:** 1 Service de Radiologie et d’Imagerie Médicale, CHU Mohammed VI, Oujda, Maroc,; 2 Service de Neurologie, CHU Mohammed VI, Oujda, Maroc

**Keywords:** Encéphalopathie de Gayet Wernicke, vomissement gravidique, vitamine B1, Gayet Wernicke’s encephalopathy, vomiting during pregnancy, vitamin B1

## Abstract

L’encéphalopathie de Wernicke est une pathologie carentielle causée par un déficit profond en thiamine (vitamine B1). Elle survient le plus souvent sur un terrain alcoolique, mais parfois elle est de diagnostic difficile et dont l’évolution en l’absence de traitement conduit à des séquelles cognitives sévères. L’imagerie par résonance magnétique est l’examen de référence permettant de confirmer le diagnostic par la présence d’hyper signaux T2 au niveau périaqueducal, des thalami, et des corps mamillaires. Nous rapportons l’observation d’une femme de 30 ans ayant des vomissements abondants lors du premier trimestre de la grossesse (hyperemesis gravidarum), à l’origine d’une encéphalopathie de Wernicke symptomatique.

## Abstract (en)

Wernicke’s encephalopathy is a deficiency disease resulting from severe thiamine deficiency (vitamin B1). It mainly occurs in alcoholic subjects, but sometimes it is difficult to diagnose. The absence of treatment leads to severe cognitive sequelae. Magnetic resonance imaging is the gold standard imaging modality to confirm the diagnosis. The diagnostic sign is T2-weighted hyperintensity in the periaqueducal, thalami and mammillary bodies. We report the case of a 30-year-old woman with abundant vomiting during the first trimester of pregnancy (hyperemesis gravidarum), causing symptomatic Wernicke’s encephalopathy.

## Introduction

L’encéphalopathie de Gayet Wernicke (EGW) est une urgence neurologique secondaire à un déficit en thiamine (vitamine B1) provoquant une atteinte du réseau hippocampo-mamillo-thalamique (circuit de Papez). Elle touche de plus la substance grise au contact de l’aqueduc de Sylvius et du quatrième ventricule. Il s’agit d’une affection neurologique centrale grave avec une mortalité accédant la 30% [1]. Si l’EGW est une complication connue de l’alcoolisme, il faut savoir qu’elle peut survenir en dehors de cette addiction [2,3]. Nous rapportons un cas d’encéphalopathie de Gayet-Wernicke compliquant des vomissements incoercibles chez une femme enceinte.

## Patient et observation

**Figure 1:**
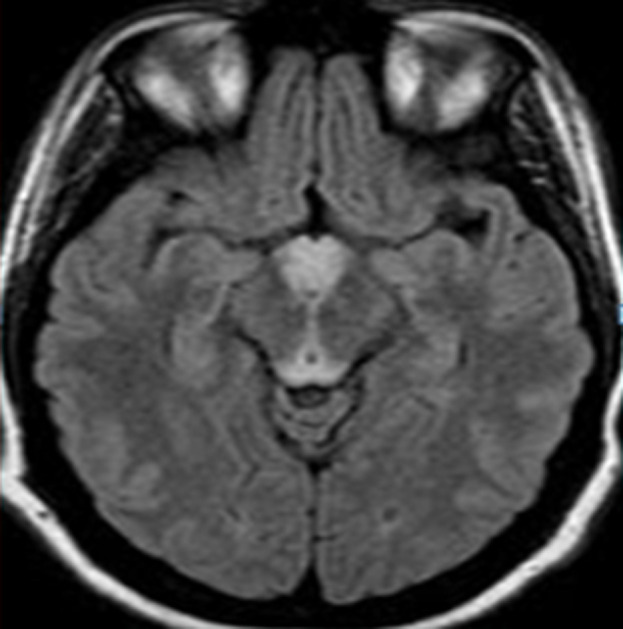
IRM encéphalique, en coupes axiale séquence FLAIR, objectivant des hyper signaux au niveau des corps mamillaires, en péri-acqueducal

**Figure 2:**
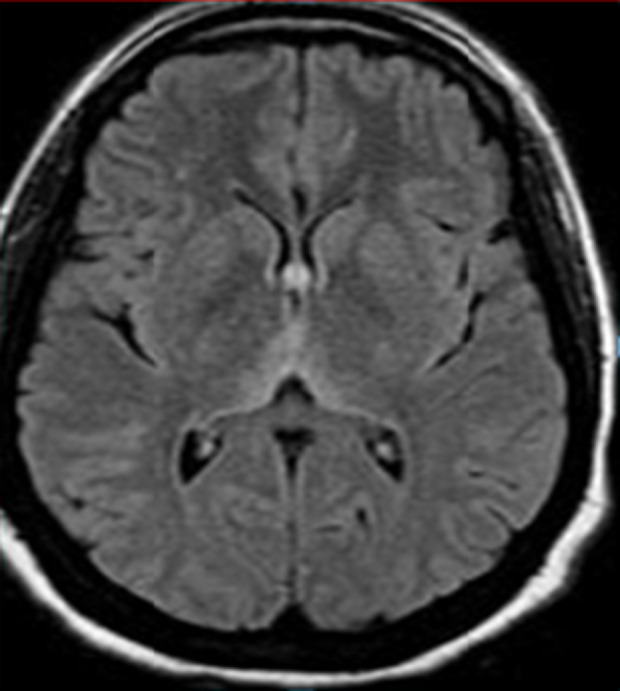
IRM encéphalique, en coupe axiale séquence FLAIR, aspect hyper intense de part et d’autre du 3^e^ ventricule

**Figure 3:**
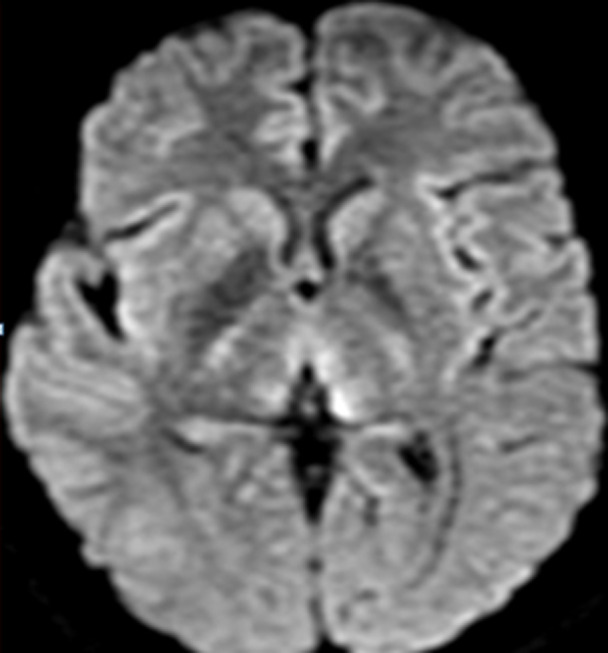
IRM encéphalique, en coupe axiale séquence de diffusion, aspect hyper intense de part et d’autre du 3^e^ ventricule

**Figure 4:**
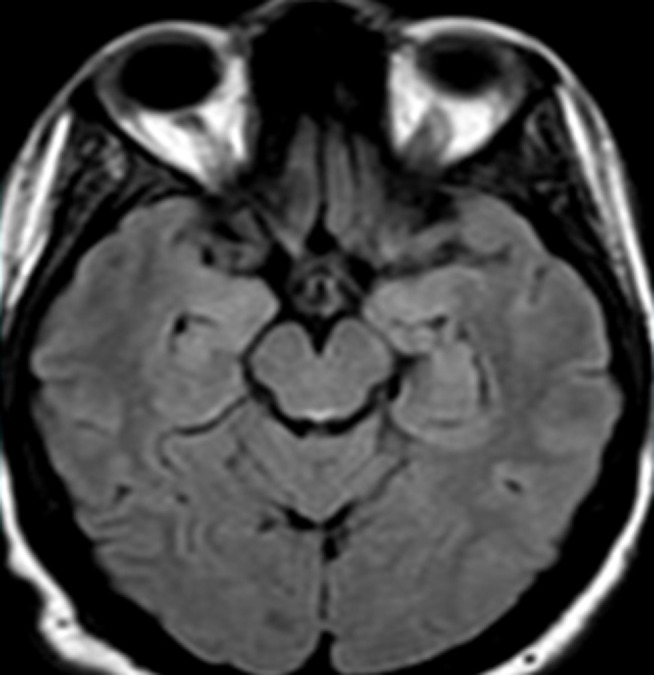
le contrôle IRM réalisé 06 mois plus tard: coupes axiales en séquence FLAIR, note une régression quasi complète des anomalies de signal

Il s’agit d’une patiente âgée de 30 ans sans antécédent (ATCD) pathologiques notables, adressée au service des urgences pour une symptomatologie neurologique faite de diplopie, syndrome d'hypertension intracrânienne (Sd d’HTIC), céphalée. La patiente était enceinte de 10 SA et rapportait des vomissements gravidiques incoercibles pris en charge de manière habituelle. La ponction lombaire était normale ainsi que le bilan biologique initial. Une image par résonance magnétique (IRM) encéphalique a été réalisée en urgence et était revenue normale. Au cours de son hospitalisation, la patiente a présenté une aggravation de son état neurologique par l’installation d’un sd confusionnel avec une importante désorientation temporo-spatiale. L’examen neurologique a révélé en outre des troubles mnésiques importants, un syndrome cérébelleux statique et cinétique, une abolition des réflexes ostéo-tendineux et une hypoesthésie essentiellement dans les membres inférieurs. Le bilan biologique a mis en évidence une cytolyse hépatique modérée, une élévation modérée des enzymes pancréatiques, une alcalose métabolique avec hypokaliémie. Une 2^e^ IRM encéphalique réalisée a montré l’apparition des hyper signaux au niveau périaqueducal, des corps mamillaires (Figure 1), des deux thalami (Figure 2, Figure 3) et autour du 3^e^ ventricule (Figure 3) très évocateurs d’une EGW. Une supplémentation en vitamine B1 parentérale (1g/24h) associée à de la vitamine B6 a été instaurée puis relayée par voie orale (vitamine B1 500 mg*2/j) jusqu’au terme de la grossesse. Deux mois plus tard, l’évolution fut marquée par une nette régression du syndrome cérébelleux statique et cinétique et de l’hypoesthésie des membres inférieurs, les troubles mnésiques par ailleurs persistaient toujours. En revanche la patiente a présenté un arrêt spontané de sa grossesse. Une IRM de contrôle faite 06 mois du début des troubles note une régression quasi complète des anomalies de signal (Figure 4).

## Discussion

L’encéphalopathie de Wernicke (ou de Wernicke-Korsakoff dans la littérature anglo-saxonne) a été décrite pour la première fois par Wernicke en 1881 chez un homme alcoolique et une femme présentant des vomissements incoercibles [4]. C’est une pathologie métabolique liée à un déficit en thiamine, le plus souvent sous-diagnostiquée avec une prévalence clinique de 0,04% à 0,13% contre 0,8% et 2,8% en anatomopathologie [5,6]. Ce sous-diagnostic est en grande partie lié à des formes cliniques trompeuses chez des patients non reconnus comme étant à risque [7,8], ainsi qu’aux fréquentes présentations atypiques. Un patient sur cinq ne présente aucun des signes cliniques de la triade classique de l’EGW; de plus ces signes sont parfois difficiles à différencier de ceux de l’intoxication éthylique aigue ou chronique, principal terrain à risque de cette pathologie dans le monde occidental [7,8]. Cette carence vitaminique B1 peut compliquer d’autres situations pathologiques telles que la malnutrition, l’anorexie mentale, la nutrition parentérale prolongée sans supplémentation thiaminique, ou encore les tumeurs gastro-intestinales, et la chimiothérapie. Chez notre patiente, l’hypovitaminose B1 était secondaire à des vomissements incoercibles dans un contexte d’hyperemesis gravidarum. L’hyperemesis gravidarum complique 0,5 a 2% des grossesses [9]. Ce sd est défini par des vomissements profus du premier trimestre de la grossesse conduisant à une perte de poids, une déshydratation extracellulaire et une alcalose métabolique avec hypokaliémie.

Une hyperthyroïdie transitoire peut être observée et participe à la sévérité de l’hypokaliémie [10]. Dans la littérature, le premier cas rattaché à l’hyperemesis gravidarum a été rapporté par Henderson en 1914 et des rares cas sporadiques ont été décrits depuis [11]. L’article de Togay-Isikay *et al*. en 2001 a permis de faire le point sur 30 cas cliniques d’encéphalopathie de Gayet-Wernicke dans un contexte d’hyperemesis gravidarum publiés entre 1968 et 2000 [12]. En 1997, Olindo *et al*. mettent en évidence l’association de ce syndrome à une myélinolyse centropontine dans le cadre de vomissements gravidiques [13]. Le diagnostic de l’EGW est avant tout clinique avec la classique triade [14] qui associe des troubles psychiques (syndrome confusionnel, apathie, bradypsychisme, hypersomnie), des troubles oculomoteurs (nystagmus horizontal ou multiple, paralysies oculomotrices par atteinte du III et du VI) et des troubles de l’équilibre, en rapport avec un syndrome vestibulaire central et un syndrome cérébelleux; cette triade n’est cependant complète que dans 30% des cas et la carence peut alors se manifester par une hypothermie, une hypotension, une tachycardie, des hallucinations, des céphalées, de la fatigue, un inconfort abdominal. Une dysarthrie, une dysphagie, une hypotonie des membres inférieurs, une hypoacousie, des myoclonies, des dyskinésies, une dystonie, une épilepsie, des troubles psychiques à type de psychose avec hallucinations auditives et délire de persécution ou de boulimie ont aussi été décrits. Une neuropathie périphérique est souvent associée, mais rarement recherchée [14]. Le syndrome de Korsakoff est décrit dans 80% des cas au décours d’une EGW, du fait des lésions du circuit hippocampo-mamillo-thalamique, avec prédominance des anomalies mamillaires [14].

En imagerie, l’IRM démontre des anomalies dans 60% des cas, ce qui implique qu’une imagerie normale n’exclut pas le diagnostic [15,16]. On peut observer dans les jours suivant l’installation des signes cliniques, des hypersignaux en T2, FLAIR et diffusion, typiques par leur localisation et leur caractère symétrique autour de l’aqueduc de Sylvius, du 3^e^ ventricule (V3), de la face médiale des thalami et surtout au niveau des tubercules mamillaires. Les séquences de diffusion objectivent des zones en hypersignal prédictives de séquelles neurologiques à long terme [17]. Par ailleurs, ces lésions prennent de façon inconstante le contraste après injection de chélate de Gadolinium [18,19]. Des localisations atypiques ont été rapportées, avec des anomalies de signal, sous la forme d’hyperintensités en T2 et possibilité de prise de contraste au niveau du vermis supérieur, de la tête des noyaux caudés et des noyaux lenticulaires, des noyaux rouges, des noyaux du nerf facial, des noyaux des nerfs abducens et des noyaux vestibulaires, ainsi qu’au niveau du corte central et précentral. Ces localisations atypiques peuvent rendre le diagnostic difficile [19]. L’IRM peut s’avérer un outil intéressant pour le diagnostic précoce des formes inhabituelles ou graves avec coma. Or, bien que très évocatrices, ces anomalies de signal ne sont pas pathognomoniques. En 1998, le travail de Antunez *et al*. avait montré que l’atteinte simultanée à type d’hypersignal T2-FLAIR des régions périaqueducales et des noyaux dorso-médians thalamiques était en faveur d’une telle encéphalopathie avec une spécificité de 93% [15].

Le diagnostic reste fondé sur les signes cliniques et avant tout, sur l’amélioration significative après traitement par thiamine. Le scanner n’a quant à lui pas fait preuve de son utilité diagnostique. Il faut garder à l’esprit les principaux diagnostics différentiels (accident vasculaire cérébral, thrombose veineuse profonde, maladie de Creutzfeldt-Jakob, syndrome de Miller-Fisher encéphalite à cytomégalovirus, lymphome). Enfin, les dosages sanguins à la recherche d’une déficience en vitamine B1 requièrent l’accès à des laboratoires spécialisés et les résultats n’y sont obtenus que tardivement, ce qui rend leur utilité limitée en pratique clinique. Pour la prise en charge et le traitement, différents protocoles ont été proposés. Il convient surtout d’introduire rapidement une vitaminothérapie B1, par voie parentérale, pour certains jusqu’à l’arrêt des vomissements et reprise d’une alimentation normale pour d’autres jusqu’à la fin de la grossesse. La réversibilité des troubles et le pronostic dépendent essentiellement de la durée des signes neurologiques avant l’introduction du traitement.

En fonction de ce délai, l’évolution peut aller de la réversibilité complète des signes cliniques et images IRM si le traitement est instauré précocement, aux séquelles motrices, au syndrome de Korsakoff (atrophie des corps mamillaires), au coma, voire au décès dans 17% des cas [20] en cas de retard diagnostique ou thérapeutique. L’évolution favorable des images n’est pas corrélée à l’évolution clinique. Si l’ophtalmoplégie régresse dans les heures suivant l’instauration du traitement, l’ataxie peut être plus longue à récupérer (séquelles dans 25% des cas) et des séquelles psycho-mnésiques plus ou moins graves peuvent être notées. Au niveau du pronostic fœtal, d’après l’article de Spruill et Kuller, l’évolution fœtale est favorable dans les différents cas publiés, lorsque la mise en route du traitement a été effectuée dans les 24 heures après l’apparition des troubles neurologiques [20].

## Conclusion

Le contexte clinique de notre patiente nous a fait suspecter en premier d’autres pathologies comme la thrombose veineuse cérébrale, un accident vasculaire cérébral, ou un autre trouble métabolique. L’IRM a facilité le diagnostic en retrouvant des hypersignaux FLAIR dans une région d’intérêt (péri-acqueducale, thalamus ou corps mamillaires). Il convient d’évoquer cette carence devant toutes manifestations neurologiques chez une patiente enceinte avec hyperemesis gravidarum afin d’éviter des séquelles irréversibles.
